# Robot-Assisted Radical Prostatectomy (RARP) Trifecta Learning Curve for Surgeons with Previous Experience in Laparoscopy

**DOI:** 10.3390/medicina60071032

**Published:** 2024-06-24

**Authors:** Altez-Fernandez Carlos, Vazquez-Martul Dario, Răzvan-Ionut Popescu, Corrales Mariela, Chantada-Abal Venancio

**Affiliations:** 1 Service of Urology, Universitary Hospital of La Coruña, 15006 A Coruña, Spain; carlos.altez@gmail.com (A.-F.C.); dario.martul@gmail.com (V.-M.D.); venancio.chantanda.abal@sergas.es (C.-A.V.); 2 Department of Urology, “Carol Davila” University of Medicine and Pharmacy, 050474 Bucharest, Romania; 3 Department of Urology, “Prof. Dr. Th. Burghele” Clinical Hospital, 061344 Bucharest, Romania; 4 Service d’Urologie, Assistance-Publique Hôpitaux de Paris, Hôpital Tenon, Sorbonne Université, 75005 Paris, France; mariela_corrales_a@hotmail.com

**Keywords:** laparoscopic prostatectomy, learning curve, trifecta, robot-assisted radical prostatectomy

## Abstract

*Background and Objectives:* Robot-assisted radical prostatectomy (RARP) is a complex surgery with a steep learning curve (LC). No clear evidence exists for how previous laparoscopic experience affects the RARP LC. We report the LC of three surgeons with vast experience in laparoscopy (more than 400 procedures), analyzing the results of functional and oncological outcomes under the “Trifecta” concept (defined as the achievement of continence, potency, and oncological control free of biochemical recurrence). *Materials and Methods:* The surgical experience of the three surgeons from September 2021 to December 2022, involving 146 RARP consecutive patients in a single institution center, was evaluated prospectively. Erectile disfunction patients were excluded. ANOVA and chi-square test were used to compare the distribution of variables between the three surgeons. LC analysis was performed using the cumulative sum control chart (CUSUM) technique to achieve trifecta. *Results:* The median age was 65.42 (±7.34); the clinical stage were T1c (68%) and T2a (32%); the biopsy grades were ISUP 1 (15.9%), ISUP 2 (47.98), and ≥ISUP 3 (35%). The median surgical time was 132.8 (±32.8), and the mean intraoperative bleeding was 186 cc (±115). Complications included the following: Clavien–Dindo I 8/146 (5.47%); II 9/146 (6.16%); and III 3/146 (2.05%). Positive margins were reported in 44/146 (30.13%). The PSA of 145/146 patients (99%) at 6 months was below 0.08. Early continence was achieved in 101/146 (69.17%), 6-month continence 126/146 (86%), early potency 51/146 (34.9%), and 6-month potency 65/146 (44%). Surgeons “a”, “b”, and “c” performed 50, 47, and 49 cases, respectively. After CUSUM analysis, the “Trifecta” LC peak was achieved at case 19 in surgeon “a”, 21 in surgeon “b”, and 20 in surgeon “c”. Conclusions: RARP LC to accomplish “Trifecta” can be significantly reduced in surgeons with previous experience in laparoscopy and be achieved at around 20 cases.

## 1. Introduction

Robotic-assisted radical prostatectomy (RARP) was first introduced in 2001 [1]. Since its inception, the technique has undergone significant development, driven by technological advances [2]. Today, RARP has become the primary surgical approach for treating localized prostate cancer in Europe [3]. Comparisons between RARP and other surgical approaches like laparoscopic or open radical prostatectomy have shown favorable outcomes for RARP, patients may benefit from reduced complications, reduced blood loss, and shorter hospital stay [4,5,6,7]. Moreover, there is evidence that suggests a higher likelihood of recovery in erectile function and continence [6,8].

A key advantage of RARP lies in its capacity to enhance the surgeon’s visual field and maneuverability through robotic assistance [9]. Utilizing three-dimensional, high-definition visualization alongside flexible robotic arms affords surgeons greater precision and dexterity during surgical procedures; this augmented visualization and range of motion facilitate meticulous dissection and tissue handling, thereby potentially enhancing functional outcomes for patients [9,10].

Understanding how surgical performance develops as surgeons gain expertise and hone their skills in specific procedures is paramount in the medical field [11]. The concept of the learning curve (LC) serves as a pivotal framework for comprehending this evolution, traditionally, the LC is evaluated by determining the minimum number of cases required for a surgeon to attain proficiency in executing an intervention [12]. However, it is important to note that while surgical time is often used as a proxy for assessing the learning curve, it may not fully capture the nuances of surgical proficiency, particularly in procedures like RARP, where functional outcomes play a crucial role [13]. As surgical metrics, clinical outcomes, and cost-effectiveness assessments are profoundly influenced by the LC, research in this domain is steadily gaining traction [14].

Given its intricate nature, RARP presents a particularly steep learning curve. Recent systematic reviews have underscored the considerable challenges associated with achieving satisfactory outcomes, often necessitating a significant caseload ranging from 70 to 350 cases [15]. While the LC of RARP has been extensively studied, there is a notable gap in the literature regarding the impact of prior laparoscopic experience on this curve [16].

Laparoscopic surgery shares some similarities with RARP in terms of the use of minimally invasive techniques and reliance on visual cues for precision. Surgeons with prior laparoscopic experience may possess certain technical skills and spatial awareness that could potentially expedite the learning process for RARP, conversely, they may also need to unlearn certain habits or techniques that are not applicable or optimal for RARP [17].

As there is a dearth of comprehensive data concerning the influence of prior laparoscopic experience on the learning curve of RARP, our study endeavors to fill this gap by examining the learning curve of RARP among three experienced laparoscopic surgeons.

This analysis will primarily focus on evaluating both functional and oncological outcomes using the ‘Trifecta’ criteria [18]. It encompasses achieving continence, potency, and oncological control while avoiding biochemical recurrence. By elucidating the learning curve of RARP in the context of laparoscopic expertise, we aim to provide valuable insights that can inform surgical training, optimize patient care, and enhance overall surgical practice.

## 2. Materials and Methods

### 2.1. Study Population

From September 2021 to December 2022, we conducted a prospective evaluation of surgical outcomes involving 146 consecutive patients who underwent robotic-assisted radical prostatectomy (RARP) under the care of three experienced laparoscopic surgeons within a single institution (University Hospital of Coruna). The experience was based on over 400 laparoscopic prostatectomies performed by those surgeons. Patients with localized prostate cancer cT1–cT2c were included. Patients with a pre-existing history of erectile dysfunction or urinary incontinence were excluded from the study. This study was approved by the Regional Ethics Committee. Written informed consent was obtained for all patients. The protocol and research respected the declaration of Helsinki principles.

### 2.2. RARP: Operative Technique

The fourth generation of the da Vinci Xi^®^ surgical system (Intuitive Surgical^®^, Sunnyvale, CA, USA) was used. The patient was placed on a 23° Trendelenburg and in a gynecological position. The procedure was performed with a transperitoneal approach, using a 0° optic (Karl Storz, Tuttlingen, Germany). The same RARP technique, as described by the Vattikuti Institute, was performed by all surgeons [19]. The choice of using a drain was left by individual surgeon’s preference.

### 2.3. Outcomes Measurements and Statistical Analysis

The evaluation encompassed the collection of demographic information, surgical data, records of surgical complications, functional outcomes, and prostate-specific antigen (PSA) follow-up at the 6-month post-surgery visit. Early continence and early potency were evaluated at 1 month. Biochemical recurrence was defined as two consecutive total serum PSA > 0.2 ng/mL. Complications were classified according to the Clavien–Dindo classification. We defined continence as no pads, except for a security pad. Potency was defined as an erection hardness scale (EHS) of 3 or 4 [20].

To compare the distribution of variables among the three surgeons, we employed statistical tests, including ANOVA and the chi-square test. The learning curve (LC) analysis was carried out using the cumulative sum control chart (CUSUM) technique to assess the achievement of the ‘Trifecta’ outcome. The SPC^®^ package, version 6.0.2.1 for statistical analysis in Microsoft Excel^®^ version 16.0 was also used.

## 3. Results

A total of 146 RARP were performed. Surgeons “a”, “b”, and “c” performed 50, 47, and 49 cases, respectively. The variables of patient age, clinical stage, ISUP grade were not significantly different between surgeons (*p* > 0.05).

Table 1 summarize the information regarding demographic data and surgical outcomes by surgeon. Mean age was 65.42 (±7.34), clinical stage was T1c (68%), T2a (32%); biopsies grade was ISUP 1 (15.9%), ISUP 2 (47.98), ≥ISUP 3 (35%). Mean surgical time and mean intraoperative bleeding were 132.8 (±32.8) and 186 cc (±115), respectively. Most patients had Clavien–Dindo II complications (6.16%). Only 5.47% had Clavien–Dindo II complications, and Clavien–Dindo III complications were rare (2.05%). Positive margins were reported in 30.13% of patients. Regarding total PSA, 99% of the mean PSA at 6 months was below 0.08 ng/mL. Only one patient presented a biochemical recurrence.

Early continence was achieved in 69.17% of patients, 6-month-continence in 86%, early potency in 34.9%, and 6-month potency in 44%.

**Figure 1:**
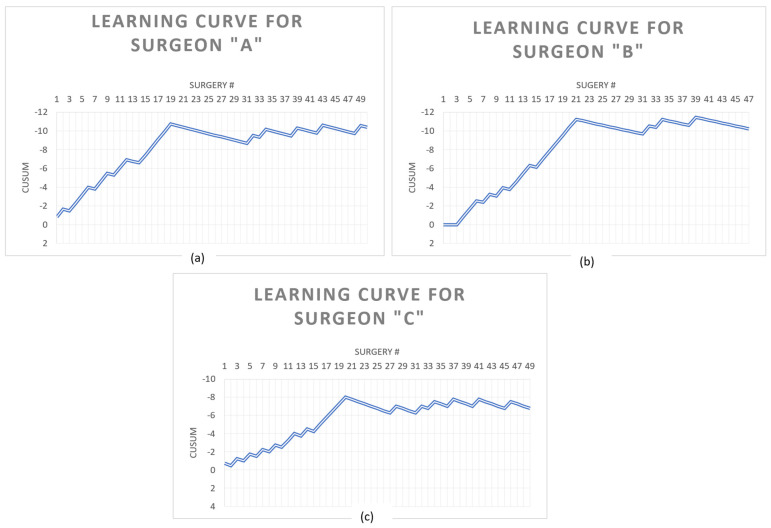
CUSUM learning curves. (**a**) CUSUM curve for surgeon “a”. (**b**) CUSUM curve for surgeon “b”. (**c**) CUSUM curve for surgeon “c”.

After CUSUM analysis, the “Trifecta” LC peak was achieved at case 19 in surgeon “a”, 21 in surgeon “b”, and 20 in surgeon “c” (Figure 1).

## 4. Discussion

The present investigation has provided invaluable insights into the intricate landscape of RARP and its associated LC, offering critical implications for surgical proficiency and comprehensive outcome assessment. Our findings strongly suggest that achieving satisfactory functional outcomes and meeting the demanding ‘Trifecta’ criteria might necessitate surgeons with prior laparoscopic experience to engage in approximately 20 cases to attain proficiency in RARP.

While the current literature primarily emphasizes operative time as the quintessential marker of the learning curve [14], we advocate for a paradigm shift towards incorporating patient-centered functional variables as more holistic indicators of surgical competence.

The assessment of surgeons’ previous experience in RARP has been notably restricted, with studies showcasing heterogeneous reporting data and a prevalent lack of comprehensive analysis considering differences in experience [14,21]. This limitation raises significant concerns regarding the comprehensive understanding of the RARP learning curve. Notably, the study of Chang et al. acknowledged the positive impact of previous laparoscopic experience on both operative time and continence rates in prostatectomies [22]. In the same direction, Ryan et al. [23] introduced the importance of a mentorship program for improving the LC of RARP; they described an analysis of a single surgeon with previous laparoscopic experience with the presence of a mentor in the initial cases, highlighting the minimal surgical complications in their series.

Tobias-Machado et al. published a retrospective study comparing their first 60 RARP with their last 60 laparoscopic prostatectomies [24]. While the authors entitled their work as questioning the real existence of a favored LC in laparoscopic experienced surgeons, they did not make a formal statistical analysis of LCs. They compared operative times, blood loss, and potency rates at 6 months. They described longer operative times for the RARP cases (243 vs. 153 min) and higher blood loss (245 vs. 202 mL) but better potency rates at 6 months (70 vs. 50%). We believe that learning curves should be analyzed formally and directed toward functional outcomes and oncologic control.

Interestingly, Dias et al. [25] have also analyzed the learning curve of laparoscopic radical prostatectomy in experienced robot-assisted surgeons. They showed that operative time could be significantly reduced, and a plateau can be reached at 40 operations. Their findings correlate well with the similarities in both surgeries that influence each of their LCs.

This study conducted a learning curve analysis utilizing the cumulative sum control chart (CUSUM) technique, a widely used method [26,27]. However, it is imperative to acknowledge the limitations inherent in CUSUM, while this mathematical model is well suited for correlating continuous variables such as operative time, its applicability to binary or nominal variables may result in misrepresented values, warranting caution in interpretation [28]. Furthermore, it is essential to note that alternative methods, like graphical inspection, split-ground analysis, and regression, offer complementary approaches for learning curve analysis in surgical procedures. These methods may provide additional insights and perspectives, potentially complementing the limitations of CUSUM and offering a more comprehensive understanding of the learning curve dynamics in complex surgeries [26,28].

Acknowledging the limitations inherent in the present study is pivotal for contextualizing our findings. Beyond the influence of laparoscopic experience, variables such as the dynamic high-volume center environment, case frequency, and intricacies of case complexity might have significantly impacted our observed learning curve. Thus, caution must be exercised in extrapolating our outcomes to low-volume centers, where differing dynamics may yield distinct learning curves. Furthermore, the exclusion of surgical margins as an outcome measure due to conflicting data necessitates further investigation to elucidate their significance in RARP outcomes [29,30]. Additionally, we want to highlight that our surgeons did not follow a pre-set mentorship program; we recognize that a mentorship program can enhance learning curves and could result in better learning curves, as shown by some studies [23,31]. Lastly, according to the new 2024 guidelines, around 16% of the patients in the study would have been selected for active surveillance instead of immediate treatment because they fall into the ISUP 1 group. Since this subgroup is relatively small, we believe that our overall study results would not be significantly impacted even if these patients had been managed differently [32].

Moving forward, a more comprehensive exploration into the multifaceted factors influencing the RARP learning curve and the delineation of efficient strategies for its progression warrants further scholarly attention. This continued research endeavor holds the promise of advancing surgical education, fostering a deeper understanding of the learning curve dynamics, and ultimately culminating in optimized patient care and elevated surgical outcomes.

## 5. Conclusions

Achieving satisfactory functional outcomes and meeting the demanding ‘Trifecta’ criteria might necessitate surgeons with prior laparoscopic experience to engage in approximately 20 cases to attain proficiency in RARP. Our study highlights the importance of incorporating patient-centered functional variables as more holistic indicators of surgical competence, moving beyond just operative time as the primary marker of the learning curve. Further studies are needed in this field.

## Figures and Tables

**Table 1:** Demographic data and surgical outcomes by surgeon.

	Total Cases	Surgeon “a”	Surgeon “b”	Surgeon “c”	p1	p2	p3
Total patients (*n*)	146	50	47	49			
Age, mean (sd)	65.42 (±7.34)	65.48 (±7.1)	65.30 (±7.69)	66.07 (±7.46)	0.75	0.42	0.38
Clinical Stage, (mean)	T1c (68%)	T1c (64%)	T1c (66%)	T1c (70%)	0.69	0.54	0.66
	≥T2a (32%)	≥T2a (36%)	≥T2a (34%)	≥T2a (30%)			
ISUP, (mean)	ISUP 1 (15.9%)	ISUP 1 (20%)	ISUP 1 (18%)	ISUP 1 (21.2%)	0.31	0.42	0.42
	ISUP 2 (47.8%)	ISUP 2 (49.5%)	ISUP 2 (44.8%)	ISUP 2 (45.8%)			
	≥ISUP 3 (35%)	≥ISUP 3 (29.5%)	≥ISUP 3 (37.2%)	≥ISUP 3 (33%)			
Mean operative time (min)	132.8 (±32.8)	122.28 (±27.02)	134 (±39.05)	156 (±25.46)	0.12	0.05	0.05
Intraoperative bleeding (mL)	186 (±115)	195.22 (±118)	166.36 (±116)	185.12 (±128)	0.16	0.34	0.18
Length of stay (h)	48 (±54.6)	48 (±27)	55.8 (±29)	30.86 (±24)	0.14	0.33	0.35
Early continence	78.60%	69%	80%	78%	0.23	0.09	0.22
6-month continence	92%	86.90%	97.50%	85%	0.16	0.18	0.33
Early potency	34%	39%	35%	42%	0.19	0.19	0.18
6-month potency	44%	48%	40%	42%	0.35	0.25	0.36

p1 compares surgeon “a” vs. surgeon “b”; p2—surgeon “b” vs. surgeon “c”; and p3—surgeon “a” vs. surgeon “c”.

## Data Availability

Data supporting the reported results are available from the authors.
